# Microsatellite status and its correlation with clinicopathological features in gastric carcinoma: insights from a retrospective study in Northern Pretoria

**DOI:** 10.3389/pore.2026.1612297

**Published:** 2026-01-23

**Authors:** Ntebogeng Kgokong, Michelle McCabe, Lucia N. Mhlongo, Thato Nkwagatse, Moshawa C. Khaba

**Affiliations:** 1 Department of Anatomical Pathology, Dr. George Mukhari Academic Laboratory, National Health Laboratory Service, Ga-Rankuwa, Gauteng, South Africa; 2 Department of Anatomical Pathology, School of Medicine, Sefako Makgatho Health Sciences University, Ga-Rankuwa, Gauteng, South Africa; 3 Department of Anatomical Pathology, Tshwane Academic Division, National Health Laboratory Service, Pretoria, Gauteng, South Africa; 4 Department of Anatomical Pathology, Faculty of Health Sciences, University of Pretoria, Pretoria, Gauteng, South Africa

**Keywords:** DNA mismatch repair, gastric carcinoma, microsatellite instability, MMR, pentaplex PCR

## Abstract

**Introduction/background:**

Gastric carcinomas (GC) are heterogeneous malignancies characterised by distinct histological and molecular subtypes. The microsatellite instability (MSI) molecular subtype, resulting from deficient DNA mismatch repair (dMMR), accounts for approximately 22% of global GC cases. Empirical evidence indicates differences in clinicopathological features, demographics, and treatment response in MSI GC compared to microsatellite stable (MSS) GC. MSI status has emerged as a potential biomarker for advanced GC, and this study aimed to determine the MSI prevalence of histopathologically confirmed GC cases at our centre.

**Method and material:**

This was a retrospective cross-sectional analysis of GC cases from 2018 to 2022, which were retrieved from the laboratory information system. DNA from these cases was isolated and assessed for MSI using a pentaplex PCR panel and confirmatory IHC on MSI-H was performed. Samples with no allelic size variation in the 5 microsatellite markers were classified as microsatellite stable (MSS), variation in 1 marker as microsatellite instability low (MSI-L), and variation in 2 or more microsatellite markers as MSI-H.

**Results:**

The study consisted of 64 cases with a MSI prevalence of 21.9% (n = 14) displaying a male predominance (n = 10; 71.4%) and a mean age of 62.7 years. Among these 14 MSI cases, 42.9% (n = 6) were classified as MSI-H with a mean age of 59.3 years. Half (n = 3) of these cases presented with upper gastrointestinal bleeding, with a majority of them diagnosed with moderately differentiated adenocarcinomas (66.7%). Microsatellite instability low was seen in 57.1% (n = 8) of the cases with a mean age of 65.3 years, and of these, patients presented with vomiting, epigastric pain and dysphagia with equal frequency at 25% (n = 2 respectively).

**Conclusion:**

The frequency of MSI cases in this study is congruent with global trends, highlighting the importance of microsatellite status in GC for understanding clinicopathological differences between MSI and MSS patients. These findings support the potential of MSI status as a biomarker.

## Introduction

Gastric adenocarcinomas (GC) represent a significant global health challenge, with over one million new cases reported annually [1]. GC is ranked as the fifth most commonly diagnosed cancer globally [1, 2] and are the third leading cause of cancer-related deaths, accounting for approximately 784,000 deaths per annum [3].

This is due to their frequent late-stage diagnoses. African patients often present with gastric adenocarcinoma at younger ages and have a higher female predilection compared to other regions which have varying prevalence across different countries [4, 5].

The aetiopathogenesis of GCs is a heterogenous process influenced by variable factors that include bacterial and viral infections such as *Helicobacter* pylori (*H. pylori*) and Epstein Barr virus (EBV), respectively [6]. However, environmental, genetic and epigenetic alterations have also been associated with GC. In lieu of this, four GC molecular subtypes have been identified by the Cancer Genome Atlas (TCGA), comprising microsatellite instability (MSI), Epstein-Barr virus (EBV), genomically stable (GS) and chromosomal instability (CIN). They are each associated with distinct genetic alterations [7].

The MSI subtype accounts for 22% of all global GC cases and is a result of pathogenic germline mutations and somatic hypermethylation in DNA mismatch repair (MMR) genes, including the MLH1, MSH2, MSH6 and PMS2 genes. These mutations occur in DNA microsatellites, which are short tandem repeat DNA sequences [8]. These microsatellite regions are prone to errors, which are detected by the MMR system and excised during replication. However, in tumour cells exhibiting the MSI phenotype, these errors go undetected and are not corrected, resulting in the propagation of further downstream mutations which then cause the initiation of gastric carcinogenesis [9]. High-frequency MSI (MSI-H) resulting from deficient DNA mismatch repair (dMMR) has emerged as a potential biomarker for advanced GC as it is identified as a separate entity from low frequency MSI (MSI-L) and microsatellite stable (MSS) GC cases due to its distinguished clinicopathological features, molecular characteristics, demographics as well as treatment response [10]. Innumerable studies have demonstrated that low-frequency MSI and MSS are clinically indistinguishable with regard to clinicopathological features and treatment responses. Consequently, both entities are thus clinically managed similarly [11, 12].

MSI-H GC has generally been characterized by distinct clinicopathological features, which include a predilection for females, an association with older ages and predominance for the intestinal subtype of the Lauren classification [10, 13, 14]. However, it is important to note that these characteristics were not uniformly investigated across different nations, as data were predominantly based on Caucasian populations. As a result, they may not be representative of all populations, particularly those of African ancestry who continue to remain underrepresented.

The diagnostic, prognostic and predictive biomarker potential of MSI showed that overall survival (OS) rates of MSI patients are consistently more improved than those of MSS patients [14]. Perioperative adjuvant chemotherapy is commonly prescribed for GC patients; however, MSI patients that receive surgery alone achieve better OS rates compared to MSI and MSS GC patients who receive adjuvant chemotherapy with fluoropyrimidines. This demonstrates the poor efficacy of chemotherapy on MSI-H GC [14, 15].

MSI-H GCs are more responsive to immune checkpoint inhibitors (ICI) showing positive correlation between MSI patients and improved survival outcomes post immunotherapy [16, 17]. This association extends beyond gastric cancer cases but is also noted in colorectal cancer as well as other malignancies characterized by genomic instability [10].

Despite the significant contributory role of MSI-H in the tumorigenesis of gastric carcinoma [13] as well as its diagnostic, predictive and prognostic biomarker potential, MSI continues to remain an untested entity in both the South African public and private healthcare systems. This in contrast to countries such as the United States through the National Comprehensive Cancer Network (NCCN) guidelines as well as several other countries following in the European Society for Medical Oncology (ESMO) guidelines [16]. This underutilized potential may negatively impact MSI-H GC patients by limiting their access to receiving effective personalized treatment options which consequently compromises patient outcomes.

This study was therefore conducted to evaluate the microsatellite status along with the causative dMMR gene(s) from GC cases in our setting with the aims of improving patient management and outcomes.

## Materials and methods

This study used a descriptive cross-sectional design which consisted of archival formalin-fixed paraffin embedded (FFPE) tissue samples of histopathologically diagnosed gastric adenocarcinoma from 01 January 2018 to 31 January 2022 at the Department of Anatomical Pathology, Dr George Mukhari Academic Laboratory, National Health Laboratory Services (NHLS), Ga-Rankuwa, South Africa.

The clinicopathological data was retrieved from the NHLS’s laboratory information system (LIS), TrackCare. Where available, the data collected included age, gender, clinical history and pathological diagnosis. Archived haematoxylin and eosin-stained and immunohistochemistry slides, and FFPE tissue blocks were retrieved from the departmental archives.

### DNA extraction and MSI PCR

The GC cases included both incisional biopsy and gastrectomy samples. The DNA was extracted from tumour samples according to manufacturers’ specifications using the QIAamp® DNA FFPE (Qiagen, Valencia, CA, USA) kit. Extracted DNA was assessed for microsatellite instability using a pentaplex PCR panel with the following set of primers: NR-27, NR-21, NR-24, BAT-25 and BAT-26, following a method detailed by Haghighi and colleagues [18]. Amplification was performed on a Veriti thermal cycler (Applied Biosystems, Foster City, CA, USA). The PCR products underwent capillary electrophoresis on the Applied Biosystems ABI 3730 automated genetic analyser, and the allele sizes were determined using Genemapper 4.0 software (Applied Biosystems, Foster City, CA, USA). Samples with no variation in either one of the five microsatellite markers were classified as MSS (microsatellite stable), variation in one marker was classified as microsatellite instability low (MSI-L) and variations in two or more markers were classified as microsatellite instability high (MSI-H).

The BAT-25 and BAT-26 microsatellite markers have been demonstrated to exhibit polymorphic variations in allele sizes among individuals of African ancestry, particularly in the South African population [19]. This genetic variability can complicate the interpretation of MSI status using PCR. To overcome this phenomenon, normal tissue (non-tumour) from GC patients in the sample population underwent MSI PCR analysis to determine whether these polymorphisms exist within the cohort. Normal tissue was obtained from GC cases where gastrectomy had been performed. These samples included resection margins that were not infiltrated by tumour or tumour deposits. DNA was then extracted from these samples and assessed for microsatellite instability.

### Immunohistochemistry (IHC)

The cases that were reported to be MSI-H underwent IHC analysis to evaluate the causative deficient MMR (dMMR) genes. The following FLEX primary monoclonal mouse antibodies were used for MLH1 (clone ES05), MSH2 (clone FE11), MSH6 (clone EP49), and PMS2 (clone EP51). Immunohistochemical analysis was performed on 4 µm sections of deparaffinized FFPE tissue sections. Heat-induced antigen retrieval (HIAR) was performed at 97 °C, and peroxidase and endogenous peroxidase activity was inhibited by the EnVision Flex Peroxidase Blocking Reagent. The antigen-antibody complexes were visualized using diaminobenzidine (DAB) as the chromogen and counterstained with Mayers hematoxylin. The stained sections were then analysed by an expert histopathologist for nuclear staining of each of the four MMR proteins. The absence of nuclear staining depicted a loss of function.

These proteins function in heterodimers, where the loss of function or degradation of one protein results in the compensatory loss of function of the other. As a result, a majority of cases report dual loss of MLH1 and PMS2 or loss of MSH2 and MSH6 (classical dMMR). Isolated loss of function can be reported in MSH6 and PMS2. The IHC dMMR results were thus reported as dMLH1/PMS2, dMSH2/MSH6, dMSH6, and dPMS2.

### Statistical analysis

Statistical analysis was performed using Graphpad Prism version 7 (GraphPad Software, La Jolla, CA, USA). Chi square and t tests were used to calculate statistical significance and make any statistical associations between microsatellite status and other clinicopathological characteristics. Statistical significance was placed at 0.05.

The study obtained ethical clearance from the Sefako Makgatho University Research Ethics Committee (SMUREC) under the number SMUREC/M/316/2024:IR.

## Results

The study included 64 gastric adenocarcinoma cases, comprising of 54 incisional biopsies and 10 cases were resection specimens (7 partial and 3 total gastrectomies). MSI positive tumours were noted in 21.9% (n = 14) of the cases. The mean age of cases with MSI positive tumours was 62.7 years and showed a male predilection (n = 10; 71.4%).

Among these fourteen MSI cases, six (42.9%) were classified as MSI-H with a mean age of 59.3 years, exhibiting a male predominance (n = 4, 66.7%). No statistical difference was observed between MSI status and age (one-way ANOVA, p = 0.59).

Fifty percent of the MSI-H cases (n = 3) displayed clinical features of upper gastrointestinal bleeding (UGIB) while patients presented with weight loss and chronic anemia at equal frequency in 16.7% (n = 1) of cases.

A majority of the tumours exhibited moderate differentiation (66.7%, n = 4) with diffuse and intestinal histopathological subtypes diagnosed at equal frequency (16.7%, n = 1) among these MSI-H tumours (Table 1).

**TABLE 1:** The clinical and demographic profiles of GC cases.

​	MSI		MSS	Statistical significance
	MSI-H	MSI-L		
Sex				
M	4 (66.7%)	6 (75%)	32 (64%)	p = 0.60
F	2 (33.3%)	2 (25%)	18 (36%)	
Mean age (years)	59.3	65.3	60.22	p = 0.59
Clinical features				
UGIB	3 (50%)	1 (12.5%)	13 (26%)	Statistical significance was not performed due to sparse cell counts
Weight loss	1 (16.7%)	-	5 (10%)	
Anaemia	1 (16.7%)	-	1 (2%)	
Epigastric pain	-	2 (25%)	11 (22%)	
Vomiting	-	2 (25%)	​	
Vomiting and weight loss	​	1 (12.5%)	2 (4%)	
Dysphagia	-	2 (25%)	3 (6%)	
Dysphagia and weight loss	​	​	1 (2%)	
Gastric outlet obstruction	-	-	5 (10%)	
*Other	-	-	4 (8%)	
Not stated	1 (16.7%)	-	5 (10%)	
Lauren’s classification				
Diffuse	1 (16.7%)	3 (12.5%)	9 (8%)	p = 0.66
Mixed	-	-	2 (4%)	
Intestinal	1 (16.7%)	1 (12.5%)	7 (14%)	
Not stated	4 (66.6%)	4 (50%)	32 (64%)	
Differentiation				
Well	-	-	12 (24%)	p = 0.5
Moderate	4 (66.7%)	4 (50%)	21 (42%)	
Poor	2 (33.3%)	4 (50%)	14 (28%)	
Not stated	-	-	3 (6%)	

*Other includes perforated peptic ulcer, pulpable abdominal mass and ascitis.

Eight (57.1%) of the cases were classified as MSI-L with a mean age of 65.3 years. Similar to the MSI-H cases, a male predominance was noted in the MSI-L cases with a male to female ratio of 3:1. Among these the most prevalent clinical features were vomiting, epigastric pain and dysphagia each presenting in a quarter (25%, n = 2) of MSI-L cases. MSI-L tumours also exhibited both moderate (50%) and poor differentiation (50%) in half (n = 4) of the cases.

The prevalence of MSS cases was 78.1% (n = 50) with a mean age of 60.22 years. MSS cases demonstrated a predilection for males with a male to female ratio of 1.8:1. The most prevalent clinical feature noted was upper gastrointestinal bleeding (26%, n = 13) followed by epigastric pain (22%, n = 11).

No statistical difference was observed between MSI status and age (one-way ANOVA, p = 0.59). Sex distribution also showed no significant difference across MSI subgroups (p = 0.6). No significant association was identified between MSI status and Lauren’s classification (Fisher’s exact test, p = 0.66). Similarly, there was no statistically significant difference in tumour differentiation across all MSI categories (Fisher’s exact test, p = 0.50).

A majority of cases (50%) demonstrated dual loss of function in MLH1/PMS2. Two cases demonstrated isolated deficiency in PMS2 (33.3%). A biopsy sample was depleted due to its small size and could therefore not undergo MMR IHC.

## Discussion

Gastric adenocarcinoma is a genetically and phenotypically heterogeneous disease with variable clinical outcomes [10, 20, 21]. Despite therapeutic advances, the lack of reliable biomarkers remains a barrier to guiding personalized therapies [20]. Precision medicine has highlighted the value of molecular classification, with microsatellite instability (MSI) recognized as a predictive biomarker for immunotherapy response and favorable prognosis [10, 20–22]. While the MSI subtype is a key component of TCGA and the Asian Cancer Research Group (ACRG) classifications, its clinical relevance remains understudied and poorly understood within the African population. To assist in addressing this knowledge gap, a retrospective study of 64 gastric adenocarcinoma cases from Ga-Rankuwa, Pretoria, was undertaken to evaluate MSI status using validated detection methods, aiming to explore its association with clinicopathological characteristics and to support the development of tailored personalized therapeutic strategies.

### Prevalence

The current study comprised a total of 64 cases of gastric adenocarcinoma in which 14 cases with MSI were identified resulting in the prevalence of 21.9%. According to literature however, the global prevalence of MSI is approximately 22% particularly in Western. However, numerous studies have reported regional variations, with MSI prevalence ranging between 5%–22% and in some instances prevalences exceeding 40% [10, 16, 23]. An Iranian comparative study of MSI in GC and its association to clinicopathological features showed prevalence rates from different countries ranging from 3.7% in Iran to 40% in India and 42% in Nigeria (Table 2) [10]. These stark differences in prevalence may be attributed to a combination of genetic, environmental, methodological, and population-specific factors. One such environmental factor is the relatively high prevalence (ranging between 60%–80%) of *H.pylori* in low and middle income countries (LMICs). *H.pylori* is an established contributory cause of GC in LMICs [24] and the highest MSI prevalence rates were noted in LMICs such as India (40%) and Nigeria (42%). It was also noted in another study that MSI prevalence was higher in black patients compared to their caucasian counterparts, this was a pattern observed in both GC and colorectal cancer (CRC) patients [25]. These disparities can be attributed to underlying genetic differences between the two race groups postulating involvement of different tumour biological mechanisms and tumourigenesis pathways.

**TABLE 2:** Immunohistochemical staining pattern in the MSI-H cases.

Case number	dMLH1	dMSH2	dMSH6	dPMS2
GC39	+	+	+	-
GC60	-	+	+	+
GC03	+	+	+	-
GC61	-	+	+	+
GC53	-	+	+	+
GC32	Biopsy tissue depleted			

Minus (−): Loss of expression. Plus (+) Expression present.

### Diagnostic methodology

There are also large variable differences in MSI prevalence observed within countries (Italy showed a prevalence of 23.5% in 2016 using the pentaplex PCR assay and an almost 10% decrease the following year with a prevalence of 14% using MMR status (Table 3) [10], indicating regional variability within national populations. These variations can also be attributed to the different MSI testing methods. There are two gold standards in MSI testing, namely IHC and PCR. However, in resource limited settings IHC remains the more economic and easily accessible method. The limitations of IHC however, are the absence of standardized reporting formats making this method prone to inter-observer differences and errors [25] as well as the fact that this method does not differentiate between high and low frequency MSI as any nuclear staining regardless of the intensity (faint, strong, slight) is considered positive. Whereas with PCR there is a clear distinction between MSI-L and MSI-H. However, the limitations with PCR analysis are the assays used. There are two assays used in PCR MSI analysis, the Bethesda panel which utilizes the analysis of two mononucleotide markers (BAT-25 and BAT-26) and three dinucleotide markers (D5S346, D2S123, and D17S250) with the option to include additional markers to increase sensitivity. The second assay is the pentaplex panel which utilizes the analysis of five mononucleotide markers (BAT-25, BAT-26, NR-21, NR-24 and NR-27) [25]. The pentaplex panel has become the preferred assay for MSI PCR analysis, largely due to the quasi-monomorphic variation range (QMVR) of the mononucleotide markers it incorporates, particularly BAT-25 and BAT-26. These markers demonstrate high stability across the general population [26] and are therefore less susceptible to allelic variation, reducing the likelihood of false-positive or false-negative results. Consistent with this, a performance evaluation of MSI PCR panels conducted on colorectal carcinoma tumors in Spain reported that the pentaplex panel exhibited comparatively better sensitivity and specificity when compared with the Bethesda panel [27]. These limitations may explain the intra-country variabilities in MSI prevalence (Table 3).

**TABLE 3:** Comparison of MSI prevalence as well as the type of testing performed stratified by region and country.

Country	Year	Prevalence	MSI diagnostic method
Current study (South Africa)	2025	21.9%	Pentaplex PCR
Iran	2023	7.5%	Pentaplex PCR
	2009	3.7%	IHC
Turkey	2021	11.6%	IHC
Korea	2019	10.3%	Pentaplex PCR
	2017	14%	15 mono and dinucleotide marker PCR
China	2021	6.9%	IHC
	2021	10.5%	5 mono and dinucleotide marker PCR
	2015	10.5%	5 dinucleotide marker PCR
Japan	2015	14.7%	15 mono and dinucleotide marker PCR
	2015	7.8%	IHC
	2012	17.7%	2 mononucleotide PCR
India	2021	40%	10 mono and dinucleotide marker PCR
Germany	2022	8.8%	PCR & IHC
​	2019	10.5%	Bethesda PCR
Italy	2017	7.5%	IHC (MMR) & pentaplex PCR
	2016	23.5%	Pentaplex PCR
Switzerland	2020	11.8%	IHC (MMR)
Russia	2021	6.9%	Pentaplex
USA	2005	19%	Bethesda PCR
	2003	16%	IHC (MMR)
Canada	2005	4.3%	8 mono and dinucleotide marker PCR
Nigeria	2020	42%	IHC – (MLH1, MSH2)

Low incidence of microsatellite instability in gastric cancers and its association with the clinicopathological characteristics: a comparative study by Talar Fateme Fooladi Talari et al. [10].

### Demographic features

Microsatellite instability in GC is known to be associated with older age groups (>65 years old) [14, 20] however, MSI-H patients were younger (59.3 years old) than the MSI-L/MSS (62.8 years old) patients in the current study. This seemingly suggests that the presence or absence of genomic instability occurs as an early event in gastric carcinogenesis particularly in black patients. A study investigating MSI in precancerous GC lesions found microsatellite instability in dysplasia and intestinal metaplasia of the stomach mucosa which are both considered early phenotypic changes preceding gastric malignancy [25]. Notably, similar patterns have been noted for MSI in CRC and prostate carcinogenesis where MSI has been detected in precancerous lesions like adenomas [28] and high-grade prostatic intraepithelial neoplasia [29] respectively.

A Nigerian study investigating MSI and its association to clinicopathological features reported a mean age of 53.3 years compared to the 51.7 years noted in the MSS cohort [30]. These findings were incongruent with those of the current study that noted a MSI patients to be older than their MSI-L/MSS counterparts. Similarly, an Iranian study also investigation associations between MSI and clinicopathological features reported that four of the total 53 patients were classified as MSI-H and of those three were all above the age of 70 except for one patient who was 23 years old [10]. It is interesting to note however that in the European studies the age of the MSI patients were predominantly above the seventh decade of life whereas the African studies including the current study had MSI mean ages in the fifth decade of life [13].

Generally, literature reports MSI-H GC to be associated with the female sex [10, 14, 20] however the findings of the current study show male predilection in both MSI and MSS cohorts with the MSI cohort showing a slightly higher male predominance. This finding correlate with those of the Nigerian study by Ahmad and Muhammed [30]. They reported a male preponderance in both the MSI and MSS group. Their findings however demonstrated a higher predominance in the MSS group with an elevated male to female ratio of 6:1 compared to the male to female ratio of 2.3:1 in the MSI group. The Iranian study by Talaria demonstrated an equal male to female distribution in their MSI patients [10].

### Histopathological features

MSI-H GC has been described to be associated with the intestinal subtype of the Lauren classification [10, 14, 15]. Conversely, this was incongruent to the findings of the current study where MSI was more associated with the diffuse subtype (28.6%) compared to the intestinal subtype (14.3%). A study conducted in Western Romania involving 67 GC cases also noted the intestinal subtype to be the most common subtype (49.3%) followed by diffuse (36.1%) with the mixed subtype being the least common (14.8%). Although the primary focus of the Romanian study was the histological and surgical analysis of GC cases and did not characterize molecular features, the distribution of histological subtypes followed the global trends [30]. Notably, the majority of tumours in the Romanian study were poorly differentiated (53.7%). This was in contrast to the findings of the current study where the majority of tumours were moderately differentiated followed by those that were poorly differentiated, a pattern noted in both MSI and MSS cohorts.

### MMR IHC

In the current study, majority of MSI cases showed concurrent loss of function in MLH1 and PMS2. This finding is congruent to what is reported in literature with regards to MSI in CRC. The loss of this heterodimer pair is the most common noted in these cases [31]. An American study reviewing the utility of a two-antibody panel approach in CRC and extraintestinal tumours found the concurrent loss of MLH1 and PMS2 to be the most common loss of expression [32]. This concurrent loss can be as a result of a germline mutation in the MLH1 gene, which is often associated with hereditary cancer syndromes particularly increased Lynch Syndrome risk [33]. Germline mutations of the MMR genes in GC have been described to display a more a frequent deficiency in MLH1 and MSH2 and less frequently isolated deficiency of MSH6 and PMS2 [34]. Somatic mutations in MMR genes have also been reported in GC patients, and in this instance the MLH1 promoter silencing has been more commonly associated with the sporadic form of GC. This further aligns with the findings of the current study which displayed a higher frequency in the loss of function of the MLH1/PMS2 heterodimer pair [34].

### Limitations

This study was limited by the relatively small cohort compared to those of previous studies. Consequently, the statistical power was reduced, affecting the ability to perform meaningful comparisons and confining the generalizability of the findings to larger populations.

In addition, the retrospective study design made it difficult to obtain complete medical records for all patients, further restricting the comprehensiveness and reliability of the analyses.

## Conclusion

The findings of this study demonstrated that clinicopathological associations made with MSI gastric cancer patients are not uniform throughout different regions and populations as confirmed in other similar GC studies. Consequently, necessitating the investigation of these associations in the South African context and within different provinces throughout the country will aim to provide awareness of the distinct features present in MSI GC patients.

Although MSI makes up less than a quarter of the GC cases it is important to recognize the essential role it has in patient outcomes and survival due to the less aggressive and more manageable nature of these malignancies. Despite the diagnostic, predictive and prognostic potential of MSI in gastric carcinomas as well as its association with effective personalized therapies, this entity remains untested during patient management in South Africa. Diagnosis of MSI in GC patients can save patients from ineffective conventional cytotoxic chemotherapy and provide them access to more effective personalized targeted immunotherapy. The findings of this study therefore advocate for the screening of genomic stability in these patients to guide treatment options and subsequently improve outcomes.

## Data Availability

The raw data supporting the conclusions of this article will be made available by the authors, without undue reservation.
